# Temporal dynamics of antibody level against Lyme disease bacteria in roe deer: Tale of a sentinel?

**DOI:** 10.1002/ece3.10414

**Published:** 2023-08-17

**Authors:** Valentin Ollivier, Rémi Choquet, Amandine Gamble, Matthieu Bastien, Benoit Combes, Emmanuelle Gilot‐Fromont, Maryline Pellerin, Jean‐Michel Gaillard, Jean‐François Lemaître, Hélène Verheyden, Thierry Boulinier

**Affiliations:** ^1^ CEFE Center of Functional and Evolutionary Ecology, UMR 5175 CNRS, University of Montpellier, EPHE, IRD, Université Paul Valéry Montpellier France; ^2^ ELIZ Entente for the Control of Zoonoses Malzéville France; ^3^ University of Toulouse, INRAE, CEFS Castanet Tolosan France; ^4^ School of Biodiversity, One Health and Veterinary Medicine University of Glasgow Glasgow UK; ^5^ Department of Public and Ecosystem Health Cornell University Ithaca New York USA; ^6^ LBBE Biometry and Evolutionary Biology Laboratory UMR5558 CNRS – University of Lyon Villeurbanne France; ^7^ Ungulate Unit, Direction of Research and Scientific Support OFB, French Biodiversity Office Gap France

**Keywords:** *Borrelia burdorgferi sensu lato*, *Capreolus capreolus*, capture–mark–recapture, disease ecology, *Ixodes ricinus*, serology, tick‐borne disease

## Abstract

Changes in the risk of exposure to infectious disease agents can be tracked through variations in antibody prevalence in vertebrate host populations. However, information on the temporal dynamics of the immune status of individuals is critical. If antibody levels persist a long time after exposure to an infectious agent, they could enable the efficient detection of the past circulation of the agent; if they persist only a short time, they could provide snap shots of recent exposure of sampled hosts. Here, we explored the temporal dynamics of seropositivity against Lyme disease agent *Borrelia burgdorferi sensu lato* (*Bbsl*) in individuals of a widespread medium‐sized mammal species, the roe deer (*Capreolus capreolus*), in France. Using a modified commercially available immunoassay we tested 1554 blood samples obtained in two wild deer populations monitored from 2010 to 2020. Using multi‐event capture‐mark‐recapture models, we estimated yearly population‐, age‐, and sex‐specific rates of seroconversion and seroreversion after accounting for imperfect detection. The yearly seroconversion rates indicated a higher level of exposure in early (2010–2013) than in late years (2014–2019) to infected tick bites in both populations, without any detectable influence of sex or age. The relatively high rates of seroreversion indicated a short‐term persistence of antibody levels against *Bbsl* in roe deer. This was confirmed by the analysis of samples collected on a set of captive individuals that were resampled several times a few weeks apart. Our findings show the potential usefulness of deer as a sentinel for tracking the risk of exposure to Lyme disease *Bbsl*, although further investigation on the details of the antibody response to *Bbsl* in this incompetent host would be useful. Our study also highlights the value of combining long‐term capture‐mark‐recapture sampling and short‐time analyses of serological data for wildlife populations exposed to infectious agents of relevance to wildlife epidemiology and human health.

## Graphical abstract

To determine whether roe deer is an interesting species to detect the risk of exposure to *Borrelia burgdorferi sensu lato* (*Bbsl*). We determined the temporal persistence of antibody levels against *Bbsl* in three roe deer populations. Among the three populations, we performed capture–mark–recapture modelling on two natural populations at an annual scale and one in monthly scale.

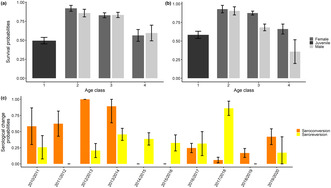

## 1 INTRODUCTION

Monitoring changes in the risk of exposure to disease agents circulating in wildlife and vectored by arthropods has become critical in the context of environmental change related to human activities (Kilpatrick & Randolph, 2012). The presence of pathogens can be tracked by detecting the agents in hosts or vectors or by indirect approaches such as the detection of antibodies in hosts (Kurtenbach et al., 1998; Rijks et al., 2019). Antibodies are molecules produced by B cells following the exposure of a vertebrate host to an antigen; they may inactivate an antigen and ensure its destruction and/or promote the immune response. Depending on the host species and the antigen, the dynamics of the antibody response may differ, leading to variable temporal persistence of detectable antibodies after exposure(s) (Benavides et al., 2017; Gamble et al., 2020; Gilbert et al., 2013; Tizard, 2017). If antibody levels persist in the long run following the exposure of individuals to an infectious agent, they could enable the efficient detection of the past circulation of the agent in an area. On the contrary, if antibody levels only persist for a short period, they could provide snapshots of the recent exposure of sampled hosts. Tracking the temporal variation in antibody levels should thus be highly relevant for the monitoring of spatio‐temporal exposure to a pathogen.

One challenge associated with serological analyses of vertebrates in the wild is that tests specifically developed for the targeted host species are often not available, nor gold standards exist to determine which individuals have circulating antibodies (i.e. are seropositive) or not (i.e. are seronegative) against a specific agent (Benavides et al., 2017; Gilbert et al., 2013). Different approaches can nevertheless be used, notably by running immuno‐assays developed for related species and using information provided by the distribution of the assay results (Garnier et al., 2017). Serological approaches are then powerful to quantify evidence of past exposure to an infectious agent, and they can be used in species that could be considered sentinels (Halliday et al., 2007). This is notably the case when a species is widespread and abundant and is highly exposed to the source of transmission of the infectious agent of interest, such as roe deer (*Capreolus capreolus*) and tick‐borne pathogens in Europe.

Lyme disease (LD) is the most widespread tick‐borne zoonosis in the northern hemisphere (Marques et al., 2021). LD is caused by a spirochete bacterium of the *Borrelia burgdorferi sensu lato* (*Bbsl*) complex, which is transmitted through the bites of infectious hard ticks of the Ixodidae family, in particular, *Ixodes ricinus* in Europe (Kurtenbach et al., 2006). This tick species has three feeding stages (larvae, nymphs and adult females) that take each a single blood meal on the host. After each meal, the tick detaches from the host to moult (larvae, nymphs), lay eggs (female adult), or die (male adult after fertilizing a female on the vertebrate host). Ticks can feed on various host species, in particular, small rodents for the first stages and mainly large vertebrates for the nymphal and adult stages (Kilpatrick et al., 2017).

At present, LD risk is assessed from data on human cases, tick density and *Bbsl* prevalence in ticks, or data on *Bbsl* (sero)prevalence in tick hosts. In both Europe and the United States, human LD cases have dramatically increased in the last decades such as from 2500 in 1990 to 35,000 in 2010 in Europe (Dumic & Severnini, 2018; Kugeler et al., 2021; Mead, 2015; Vandekerckhove et al., 2019). This trend is broadly attributed to changes in a range of ecological factors leading to increasing tick–host interactions (changes in land use, climate change, increased habitat fragmentation, and changes in vegetation and host community structure), on top of an increase in disease awareness (Kilpatrick et al., 2017). However, trends at large geographic scales remain difficult to assess. Surveillance systems for LD are heterogeneous across countries, with human LD being classified as a notifiable disease in some countries, while in others, like in France, the data collection is based on both a network of volunteer doctors and hospitalization data (Fu et al., 2021; Smith et al., 2006; Van Den Wijngaard et al., 2017; Vandenesch et al., 2014). LD risk can be also assessed by tick sampling using cloth dragging to track apparent tick densities followed by polymerase chain reaction (PCR) to search for *Bbsl* DNA in ticks, but this approach has limits. Cloth‐dragging has a low sampling efficiency (Nyrhilä et al., 2020) and produces high uncertainties in tick abundances (Bord et al., 2014), depending on the vegetation composition and weather conditions (Salomon et al., 2020). In addition, tick collection for quantifying reliably *Bbsl* infection rates can be a time‐consuming process because large samples are required when prevalence is low and local availability of ticks can vary greatly (Estrada‐Peña et al., 2021; Healy & Bourke, 2004, 2008). Alternatively, the risk of exposure to LD can be assessed from animal sentinels, including domestic and wild mammals. Surveys of domestic dogs can for instance be conducted at veterinary practices (Duncan et al., 2005; Smith et al., 2012), but this could induce a sampling bias because the canine population with the highest risk of tick exposure could be animals that receive no veterinary care (Watson et al., 2017). In North America, the white‐tailed deer (*Odocoileus virginianus*) has been considered an efficient sentinel for the risk of exposure to LD (Adetunji et al., 2016; Gallivan et al., 1998; Gill et al., 1994; Magnarelli et al., 2010; Raizman et al., 2013). In Europe, a species with similar ecology, the roe deer (*Capreolus capreolus*), has less often been the subject of serological studies, possibly because of its known innate immune system ability to clear *Borrelia* infection by activating the alternative complement pathway, which might affect antibodies production after exposure (Bhide et al., 2005; Telford et al., 1988).

The roe deer has long been proposed to serve as a sentinel for tick‐borne pathogens, such as the tick‐borne encephalitis virus (Gerth et al., 1995; Rijks et al., 2019) because it is widely distributed and highly abundant all over Europe (Andersen et al., 2000). Access to roe deer samples is relatively easy because more than 1,800,000 individuals are hunted each year in Europe (Linnell et al., 2020). Moreover, roe deer can be highly infested by ticks (Vor et al., 2010) and have small and stable home ranges (Morellet et al., 2013). The high spatial fidelity of roe deer could make seroprevalence against tick‐borne pathogens in this species a reliable proxy of the local risk of exposure to infected ticks. Regarding the antibody response of roe deer to *Bbsl*, in contrast to white‐tailed deer for which specific immune responses to *Borrelia burgdorferi sensu stricto* strains have been reported (Luttrell et al., 1994), few studies have addressed this issue (Bhide et al., 2004). In roe deer, the lysing effect of the complement on the bacteria *Bbsl* has been reported as an efficient early innate immune defense (Bhide et al., 2005), with potential effects on the buildup of an antibody response. Nevertheless, the presence of anti‐*Bbsl* antibodies in that species has been reported, with high seroprevalence in tick‐infected areas (Alonso et al., 2012; Juřicová & Hubálek, 2009; Pato et al., 2013; Webster & Frandsen, 1994). Further, in a cross‐sectional study conducted in Spain, seroprevalence was higher in spring than in winter months, and in animals with higher tick burden (Pato et al., 2013). However, little information is available on potential temporal change in the serological status of individual roe deer against *Bbsl*, despite the usefulness of such information for interpreting seroprevalence data and interactions between the host immune defense and infection, as reported for instance in primates (Embers et al., 2012; Woudenberg et al., 2020). In wild settings, the longitudinal sampling of individuals for epidemiological purposes is rarely available, and when capture–recapture data are available, individuals are often not captured on all sampling occasions, which requires the use of a modelling approach for estimating rates of change of serological status by accounting for imperfect detection (Conn & Cooch, 2009; Gamble et al., 2020).

The aim of this study was to explore the dynamics of anti‐*Bbsl* serological status in roe deer by using longitudinal data. To do so, we analyzed roe deer sera from two wild‐living populations and one captive population in France. Using multi‐event capture–mark–recapture (CMR) models (Pradel, 2005), we analyzed data collected between 2010 and 2020 in a longitudinal monitoring of the two wild roe deer populations to estimate annual rates of seroconversion (i.e. the proportion of individuals switching from seronegative to seropositive) and seroreversion (i.e. the proportion of individuals switching from seropositive to seronegative). In particular, we investigated whether the positive serological status of individuals is transient when individuals are not re‐exposed to the bacteria, which could be revealed by high seroreversion rates. Further, we predicted that annual seroconversion and seroreversion rates could vary among years and between populations due to variable immune responsiveness, with possible effects of the local context (Cheynel et al., 2017), sex and age classes in relation to physiology (Jégo et al., 2014) and behaviour (Andersen et al., 2000). Chizé population would be expected to show higher rates of seroconversion because winters are milder than Trois‐Fontaines and could lead to higher exposure to ticks at that time. We also expected a higher seroconversion rate for males because previous studies have shown a higher exposure of males than females roe deer to ticks (Kiffner et al., 2010; Vázquez et al., 2011; Vor et al., 2010). In addition, we expected that adult roe deer could have a higher seroconversion rate because previous results on these populations had shown an increase in inflammatory markers with age (Cheynel et al., 2017). Overall, we also expected high seroreversion rates as roe deer are known for not being competent for *Bbsl* (Jaenson & Tälleklint, 1992; Telford et al., 1988), which might be associated with a low persistence of antibody level following exposure or re‐exposure to *Bbsl* and strong inactivation by proteins of the complement system (Bhide et al., 2004, 2005).

Finally, we investigated the within‐year temporal rates of change in the serological status in a few captive roe deer that could be repeatedly sampled over several months to assess short‐term antibody persistence. The results were then used to discuss the potential for roe deer to be used as a sentinel for the monitoring of LD exposure risk in time and space.

## 2 MATERIALS AND METHODS

### 2.1 Study populations and sampling

**FIGURE 1:**
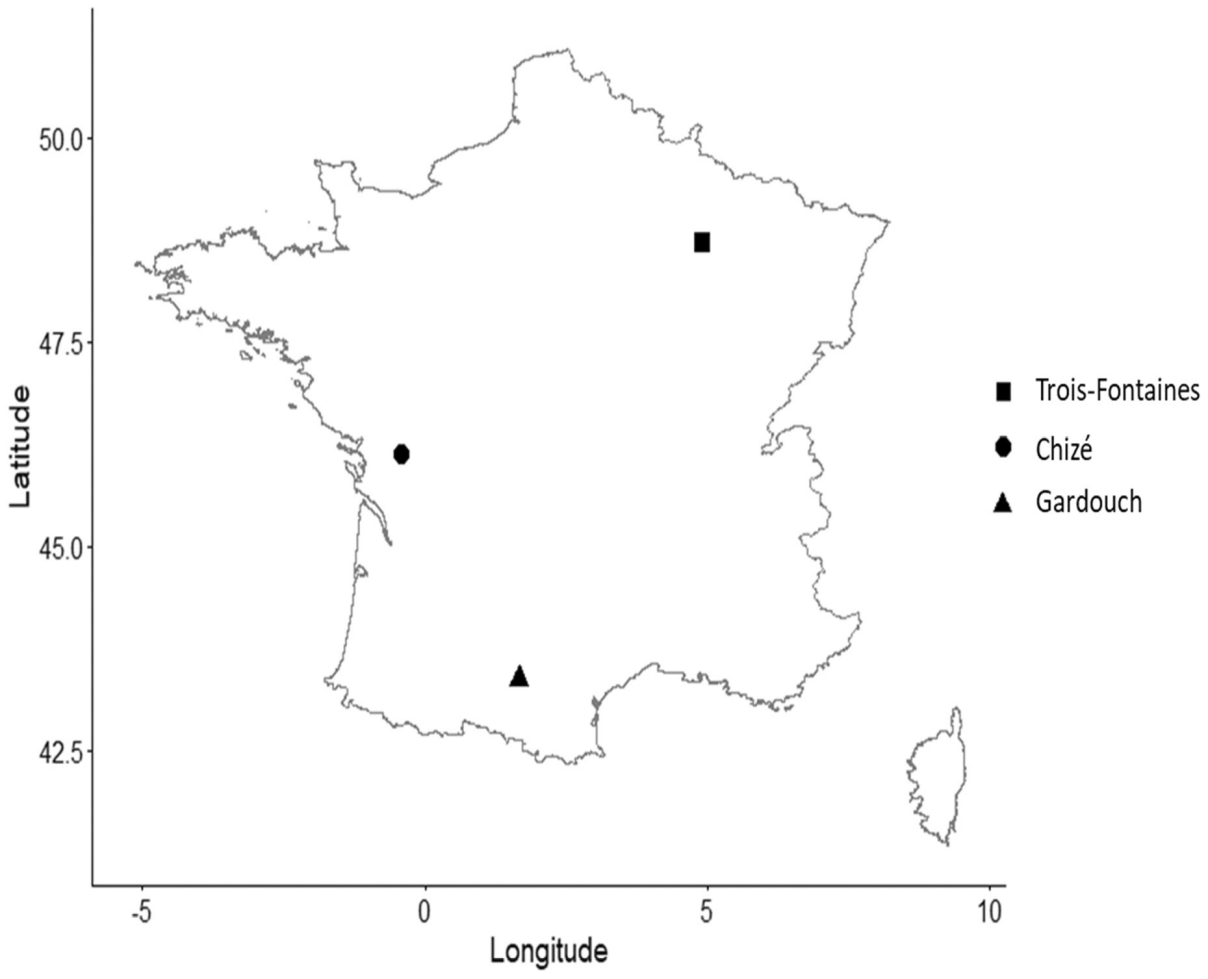
Location of the study sites in France. Trois‐Fontaines (TF) and Chizé (CH) are sites where a long‐term capture–mark–recapture of roe deer has been conducted and blood samples were available each year between 2010 and 2020. Gardouch (GA) is the site where four roe deer individuals in enclosures could be sampled repeatedly over a series of months.

The study was conducted on three roe deer populations intensively monitored in France: two wild‐living populations living in enclosed forests (Trois‐Fontaines: TF and Chizé: CH) and a captive population kept in an experimental station (Gardouch: GA) (Figure 1). In the forest populations, captures have been conducted each year to gather information and samples on individual deer over their lifetime. Annual adult survival was high (0.85 in males and 0.92 in females; Gaillard et al., 1993) because no or little hunting occurred in those sites.

Trois‐Fontaines is an enclosed forest of 1360 ha in the north‐east of France (48.71°N, 4.92°W) managed by the Office Français de la Biodiversité as a field site for investigations and experimentations. It is situated in an area with a continental climate characterized by cold winters and hot, dry summers. CH is an enclosed forest of 2614 ha located in the western part of France (46.15°N, −0.42°W) managed as a reserve by the Office Français de la Biodiversité. The climate is temperate oceanic with cold or mild winters and mild and rainy summers. Every year and for each site, 10–15 days of capture were organized between December and March at the same time. Individuals were captured using drive beaters and nets once a year (see Gaillard et al., 1993, for further details about the capture protocol). All captured animals were sexed, weighed and inspected for marks or newly marked with ear tags and numbered before being released. The age was known for most of the captured individuals because they had been captured first either a few days after birth in the spring or at the onset of their first winter when less than 1‐year‐old roe deer can be reliably identified by the presence of milk teeth. Each year, 100–250 roe deer were caught depending on the year and population. Since December 2009, a blood sample was taken from the jugular vein for all known‐aged roe deer to conduct immunological analyses. Serum was extracted from the samples and stored at −20°C until analysis (see Cheynel et al., 2017; Gilot‐Fromont et al., 2012 for further details).

Roe deer raised in the GA experimental station was sampled to investigate short‐term (within a year) dynamics of anti‐*Bbsl* antibody levels. GA site is an experimental platform of 20 ha located in south‐western France (43.37°N, 1.67°E) where 9 tame roe deer were raised in small groups (1–4 individuals) in enclosures of 0.2–0.5 ha fed with pelleted food. Roe deer in GA were all of known age and were known to be highly exposed to ticks (Wongnak et al., 2022). Tame roe deer raised in the GA site were routinely visited and petted by trainers. From October 2020 to July 2021, blood droplets were sampled from four females on blotting paper following a gentle needle prick on the ear. These four females have been sampled repeatedly at least seven times over several months in 2020–2021. The blotting paper was stored at −20°C until elution.

### 2.2 Immunological assays and determination of serological status

For GA samples, the blotting papers were first eluted in DILBUF buffer (Borrelia + VlsE IgG ELISA Kit; IBL International) from the ELISA kit. Elution was implemented overnight by cutting a 1 cm^2^ piece of blotting paper and putting it in a 2 mL tube filled with the buffer. We then used a modified commercial anti‐*Bbsl* enzyme‐linked immunosorbent assay (ELISA) kit (Borrelia + VlsE IgG ELISA Kit; IBL International) to quantify antibody levels against *Bbsl* in the serum (CH and TF) and blotting paper (GA) samples of roe deer to determine their serological status at the time of sampling. The principle of replacing the conjugated antibodies in a commercial kit with relevant conjugated antibodies for a species of interest is not new (e.g. Staszewski et al., 2007) but needs to be adapted to a given set‐up. In the case of the current study, peroxidase‐conjugated anti‐human IgG antibodies were replaced with peroxidase‐labelled anti‐deer IgG antibodies (peroxidase‐labelled antibodies to deer IgG (H+L); 04‐31‐06, KPL). Antibody levels were expressed as optical density (OD) obtained from a spectrophotometer reading at 450 nm.

**FIGURE 2:**
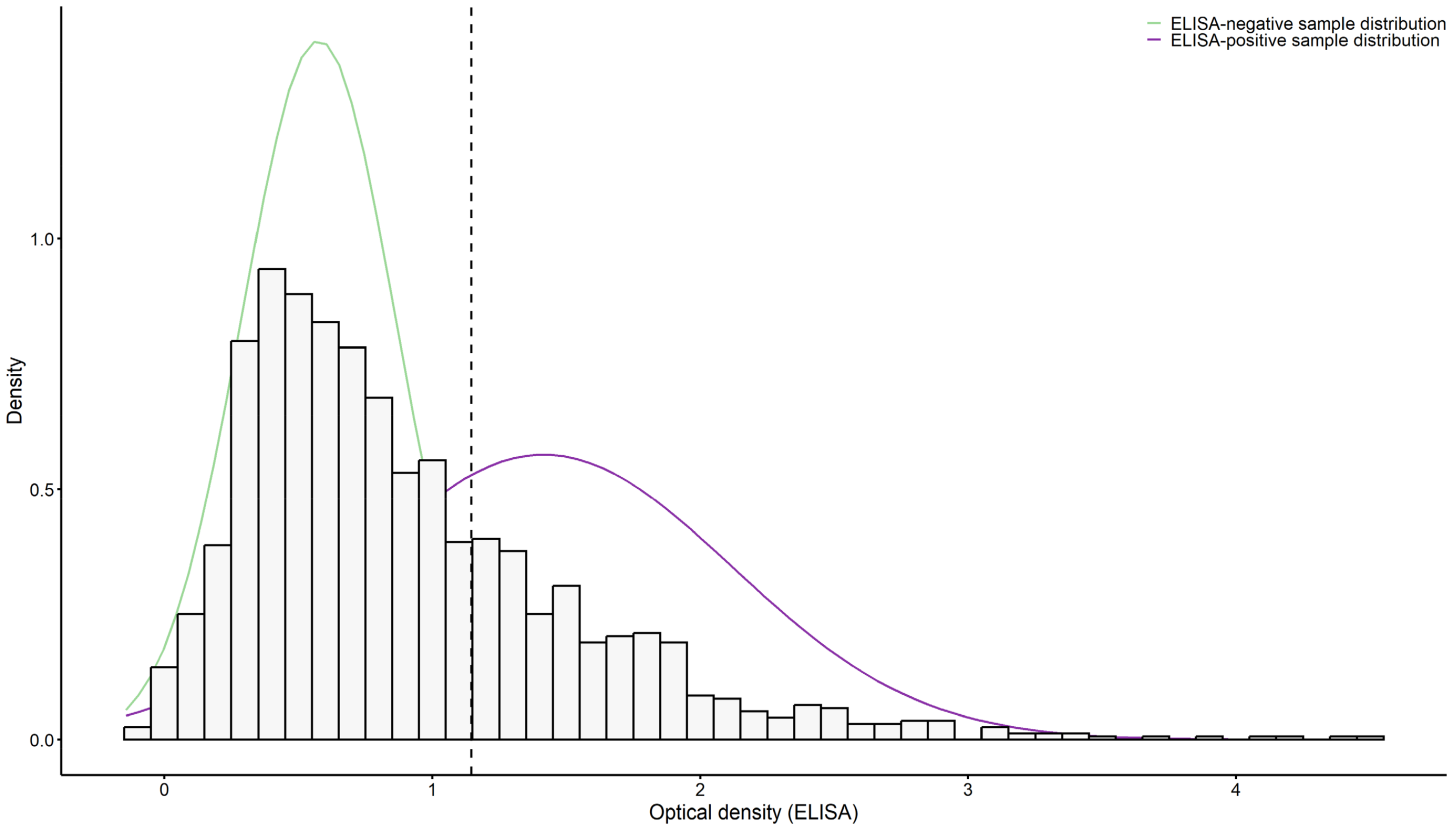
Distribution of optical density (OD) measures at 450 nm of the modified anti‐*Bbsl* ELISA in roe deer sampled at TF and CH. The distribution is bimodal, with low values corresponding to seronegative sera samples and high values to seropositive sera samples. The histogram presents the normalized counts of individuals, and the curves correspond to the probability density function of two fitted normal distributions. The dotted line represents the threshold of seropositivity, defined as the mean value of the negative distribution plus 2 times its standard deviation: 1.146 for TF and for CH.

The serological status was inferred using the distribution of observed ODs (Garnier et al., 2017). Analyses were performed using R software version 4.2 with the ‘mixtool’ (Benaglia et al., 2009) and ‘MASS’ package (Venables & Ripley, 2002). Although, we expected a bimodal distribution of ODs (seronegative vs. seropositive results), we also tested whether a single distribution (no possibility to differentiate between seronegative and seropositive) or a three‐Gaussian distribution (three groups due to individual differences in responsiveness) better fitted the data. Moreover, individual differences may imply the bimodal problem is not enough to describe the process, thus we tested for three distributions. Therefore, mixture models of one versus two versus three Gaussian distributions were first fitted to the distribution of sample ODs and compared by the Akaike information criterion value (AIC) (Burnham & Anderson, 2004). The seropositivity threshold was inferred from the mixture model with the lowest AIC. In bimodal distributions of OD values, low values were assumed to correspond to seronegative samples and high OD values to seropositive samples (see Garnier et al., 2017 for methodological details). The threshold for an individual to be considered seropositive was set by the OD value of the right bound of the 95% confidence seronegative interval (Garnier et al., 2017). So, the OD‐positive threshold was determined by adding two standard deviations (1.96) of the mean OD value to the mean OD value of the distribution corresponding to the negative individuals (Figure 2). Individuals were considered seropositive with no error when their OD value was greater than that threshold, whereas individuals with an OD value lower than that threshold were considered seronegative with possible misidentification. We analysed all samples together to determine a common threshold of seropositivity.

The seropositive threshold was further checked and the results were qualitatively robust to the use of threshold obtained from both populations. To do this, we checked the specificity of the ELISA assay. We performed immunoblots (Lyme IgG + VlsE Western Blot; EuroImmun) on 28 of the serum samples with values spread over the range of OD values. The test was performed to confirm that for values of OD above the threshold, antibodies reacted to specific markers of *Bbsl* surface (VlsE, OspA, OspC, etc.). A positive result was characterized by the presence of at least one band on the VlsE marker. As the assay was designed for human serum, we replaced the peroxidase‐conjugated anti‐human IgG antibodies with peroxidase‐conjugated anti‐deer IgG antibodies (peroxidase‐labelled antibodies to deer IgG (H+L); 04–31‐06, KPL) and the substrate of kit by a TMB (3,3′,5,5′‐tétraméthylbenzidine) solution (ELISA 1‐Step™ Ultra TMB, ThermoFisher).

Finally, we checked the correlation between serum and blotting papers. To do this, we compared the annual OD measurement from blood on blotting paper and sera from the same individuals. These individuals were already collected for another purpose and the correlation was found to be relatively strong (*r* ^2^ = .92, *n* = 20). The stability of the ELISA result was evaluated by repeating OD measurements of serum (*r* ^2^ = .98, *n* = 20) and blotting paper samples (*r* ^2^ = .96, *n* = 20) from those individuals.

### 2.3 Modelling annual variation in roe deer serological status from capture–mark–recapture data

In order to estimate the seroconversion and seroreversion rates while accounting for factors affecting the history of capture–recapture of individuals along the series of (yearly) sampling occasions and the fact that data on the serological status of individuals is thus not available for all years, we used a multi‐event CMR approach (Pradel, 2005; Schwarz et al., 1993) implemented in E‐SURGE (Choquet, Lebreton, et al., 2009; Choquet, Rouan, & Pradel, 2009) for TF and CH populations. Such models have been developed to deal with the analysis of capture–recapture data that combine a between‐state transition process (biological process) and an observation process (events) (Pradel, 2005). We started by building a table with the capture–recapture history of each individual and its serological status, with for each year (i.e. sampling occasion) whether the individual was not captured or observed (‘0’), or captured and/or observed and its serological status tested (‘1’ if not tested by ELISA, ‘2’ if tested seronegative, ‘3’ if tested seropositive).

We then proceeded with the CMR analysis. First, in order to check the fit of general capture–recapture models to the data and because there is currently no specific test to assess the goodness‐of‐fit (GOF) of multi‐event models, we tested GOF for the single‐state Cormack‐Jolly‐Seber (CJS) model (Cormack, 1964; Jolly, 1965; Seber, 1965), thus without considering the serological information. GOF tests were performed independently for each population. Using these tests, we explored a potential effect of juvenile mortality: young individuals (‘new individuals’) have a lower expectation of being re‐observed in the future, compared to individuals captured at the same occasion that had been captured previously (‘old individuals’). To do so, we applied the ‘3G.SR’ test procedure separately on juveniles and individuals ≥2 years old. Additionally, we tested a trap‐dependence effect by the ‘2.CT’ test to determine if individuals not captured before at a given sampling occasion had the same probability of being recaptured at the next occasion as currently captured individuals (Pradel, 2005). To do so, we performed the 2.CT test separately on juveniles and individuals ≥2 years old. To perform GOF tests, we used the stand‐alone U‐CARE software (Choquet, Lebreton, et al., 2009; Choquet, Rouan, & Pradel, 2009).

To explore the dynamics of the serological status of roe deer (f), we used a multi‐event model (Schwarz et al., 1993) with three states (seronegative, seropositive and dead). In our CMR modelling, the structure of the multi‐event models consisted of three main components (see Figures S1 and S2 for details). The first component is a matrix composed of the initial proportions of individuals in each serological status (π). The second component is a matrix of survival probabilities (s) and survival–conditional serological transitions (ψ). In the serological transitions (ψ), we differentiated the annual rates of seroconversion (ψ_seroconversion_) and seroreversion (ψ_seroreversion_) from the two populations (see Figure S2 for details). Finally, the third component is an observation matrix including three conditional sub‐processes: (i) the probability of recapture of individuals (p), (ii) whether the ELISA test was performed at the time (year) of the capture of the individual (σ) and (iii) the possible error on the serological status (e) (i.e. ELISA test plus the determination of the status). In our CMR models, we assumed that there is no error in the assignment of the positive (tested seropositive) serological status of individuals. Conversely, the seronegative assignment (tested seronegative) may be subject to error, in that case, individuals can be either seropositive or seronegative.

We defined a general model as described below. Following the result of the ‘3G.SR’ test and previous works (Choquet et al., 2011; Gaillard et al., 1993), four age classes (a4) at capture were distinguished to account for age‐dependent survival previously reported: ≤1‐year‐old (juveniles), 1 < year‐old ≤ 3, 3 < year‐old ≤ 9, >9‐year‐old (see Table S3 for details). In addition, the interaction between the sex and the age class 2, 3 and 4 was implemented in the survival matrix given by the result of the 3G.SR test and previous works (Choquet et al., 2011; Gaillard et al., 1993) consistently retrieved higher adult, but not juvenile survival of females compared to males. The location (pop) was also implemented because the survival probabilities were different in TF and CH populations in previous work (Gaillard et al., 1993). Concerning the observation matrices, for recaptures, we implemented an interaction of two age classes (a2) (juveniles vs. adults) according to the year (t) and location (pop) because the capture probability was expected to be higher in juveniles than in adults (Gaillard et al., 1997) and the field effort varied according to the years. The ELISA test error was implemented as constant (c). The general model can be written as:

$$\pi \left(\mathrm{pop}.a4.\mathrm{sex}\right),s\left(\mathrm{pop}.\mathrm{sex}.f.a4.t\right),\psi \;\left(\mathrm{pop}.a4.\mathrm{sex}.f.t\right),p\left(\mathrm{pop}.a2.t\right),\sigma \;\left(t\right),e\left(c\right)$$

Given the complex structure of the general model, we selected a suitable model in three steps. The first model selection step compared models with different sets of initial proportions of serological status (e.g. similar or not between age classes). The second model selection step was then performed to compare models considering or not an association between the individual serological status and survival. Finally, a third model selection step was conducted to explore potential sources of variation in the transitions of the serological status of individuals, considering the possible association with age and/or sex and/or time and/or site. Thus, we selected the best model after the succession of three steps of model selection. For each step, the most parsimonious model that best fitted the data was selected using the stand‐alone E‐SURGE software (Choquet, Lebreton, et al., 2009; Choquet, Rouan, & Pradel, 2009). The models were evaluated and compared using an information‐theoretic approach based on the Akaike information criterion (AIC) adjusted for small sample sizes (AICc) (Burnham & Anderson, 2004). The model construction is served in E‐SURGE by two modules called GEPAT (GEnerator of PATtern of elementary matrices) and GEMACO (Generator of Matrices of Constraints). The model with the lowest AICc value was selected. Details on the implementation of the analyses are provided in Tables S4 and S5 and Figure S1.

## 3 RESULTS

### 3.1 Seroprevalence in forest populations

**FIGURE 3:**
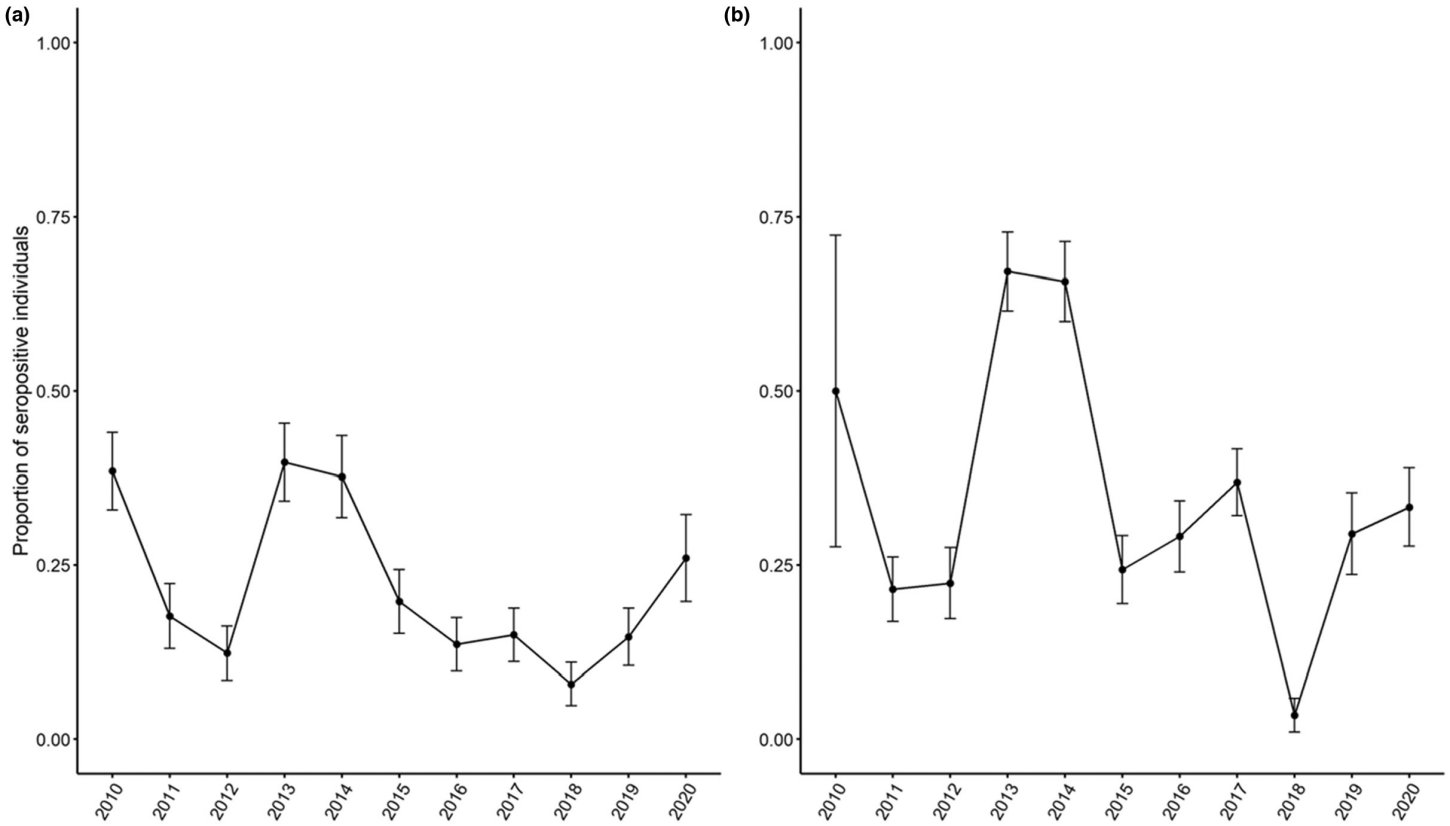
Change in roe deer seroprevalence against *Bbsl* between 2010 and 2020 for individuals of TF (a) and CH (b). The error bars in black represent the standard error of the mean.

A total of 1554 blood samples obtained from 899 different individuals were analyzed with the ELISA test (sample structure detailed in the Tables S1 and S2) including 470 individuals for TF and 429 individuals for CH. An average of 1.87 (SE: 0.066) captures per individual for TF and 1.96 (SE: 0.068) captures per individual for CH were obtained with several individuals captured only once, but many captured four times or more (Figure S3). The mixture model on the OD values with two normal distributions was retained compared to a model with one normal or three normal distributions (AIC value: 2657.820 for the retained model, with ΔAIC = 465.315 and 4, respectively, for the other two models). The distribution of OD values could be fitted to a mixture of two normal distributions with partial overlap, showing some uncertainty in the serological status of a fair proportion of individuals at sampling occasions (Figure 2). Given the distribution of OD values, the seropositivity threshold was 1.146 (Figure 2). The results of 19 western blots showed that all individuals with an OD value above 1.146 were positive for *Bbsl* with a specificity of the test equal to 84.21%. The annual proportion of seropositive individuals varied between 0.081 (SE: 0.032) and 0.400 (SE: 0.061) at TF (Figure 3a) and between 0.030 (SE: 0.022) and 0.671 (SE: 0.063) at CH (Figure 3b). The highest values were observed early during the study period, in 2013 and 2014, in both populations.

### 3.2 Goodness‐of‐fit tests

We observed a juvenile effect on survival rate in both populations (for TF, *p*‐value: .001; for CH, *p*‐value: .010; Table 1), in accordance with what was expected for that species (Gaillard et al., 1993). A trap‐dependence effect was also detected for juveniles in TF (for TF, *p*‐value: .028), with less evidence for CH (*p*‐value: .549; Table 1). Conversely, no trap‐dependence was detected in adults in both populations (for TF, *p*‐value: .965; for CH, *p*‐value: .600; Table 1). However, an age effect on capture was used in our general model in accordance with what is known for that species (Gaillard et al., 1993).

**TABLE 1:** Goodness‐of‐fit tests of capture–recapture modelling, with results of the ‘3G.SR’ and ‘2.CT’ tests for TF (a) and CH (b) showing evidence of juvenile mortality in both populations and a trap dependence effect only in the TF population.

Age	Test	*χ* ^2^	df	*p*‐value
**(a)**				
Juveniles	3G.SR	31.761	8	<.001*
	2.CT	17.341	8	.028*
Adults	3G.SR	1.32	4	.857
	2.CT	1.41	6	.965
**(b)**				
Juveniles	3G.SR	21.590	9	.010*
	2.CT	6.870	8	.549
Adults	3G.SR	4.329	8	.826
	2.CT	4.569	6	.600

*Note*: The 3G.SR test evaluated potential juvenile mortality by considering the hypothesis of no difference in annual survival probability between individuals newly captured as opposed to individuals previously captured. The 2.CT test evaluated a potential trap effect on the probability of recapture by considering the null hypothesis of no difference in the probability of being recaptured at a given sampling occasion, conditional on presence at that occasion and the previous one. In addition, the significant *p*‐values are denoted by ‘*’. Italics values defines the best model in selection.

### 3.3 Model selection

After the three steps of model selection, the model retained was

$$\pi \left(\text{a4}\right),s\left(\mathrm{pop}.\mathrm{sex}.a4.t\right),\psi \;\left(\mathrm{pop}.f.t\right),p\left(\mathrm{pop}.a2.t\right),\sigma \;\left(t\right),e\left(c\right)$$

During these three model selection steps, we eliminated the effect of population and sex on ‘π’, the effect of the serological status on ‘s’ and the effect of age and sex on ‘ѱ’. The QAIC value of the best model and that of the model including only a time effect on transition in serological status (ψ (f.t)) was close (ΔQAIC <2), but very similar in terms of parameter estimates (see Table 2 and Table S4 for details). The best model considered included an age effect on the initial status proportions (π), and effects of population, age and sex but not of the serological status on survival (s). In addition, transition probabilities between serological status (ψ) included a time variation. The recapture probabilities (p) included age and time effects, while the ELISA test probabilities (σ) were time dependent. For that model, the average recapture rate was lower in TF for juveniles (0.450, SE: 0.050) and adults (0.432, SE: 0.025) than in CH (juveniles: 0.630, SE: 0.054; adults: 0.512, SE: 0.025).

**TABLE 2:** Ranking of the five best multi‐event models of the serological changes selected for the population‐specific analyses of the patterns of rates of seroconversion and seroreversion.

Model parameterization on serological transition probabilities					
Transition	Id parameters	Deviance	QAIC	QAICc	∆QAICc
ψ = f.(t + pop)	*92*	*6065.4667*	*6249.4667*	*6259.738*	‐
ψ = f.t	90	6070.0433	6250.0433	6259.8634	0.1254
ψ = f.(t + a4 + pop)	90	6054.2014	6250.2014	6261.8905	2.1525
ψ = f.(t + sex + pop)	98	6062.3351	6250.3351	6261.9683	2.2303
ψ = f.(t + sex + pop)	94	6067.7355	6251.7355	6262.0068	2.2688

*Note*: Each model corresponds to a given pattern of variation of parameters associated with the probabilities of transition. Additive effects are denoted by a plus symbol (+) and interactive effects by a dot (.). Italics values defines the best model in selection.

### 3.4 Annual survival

**FIGURE 4:**
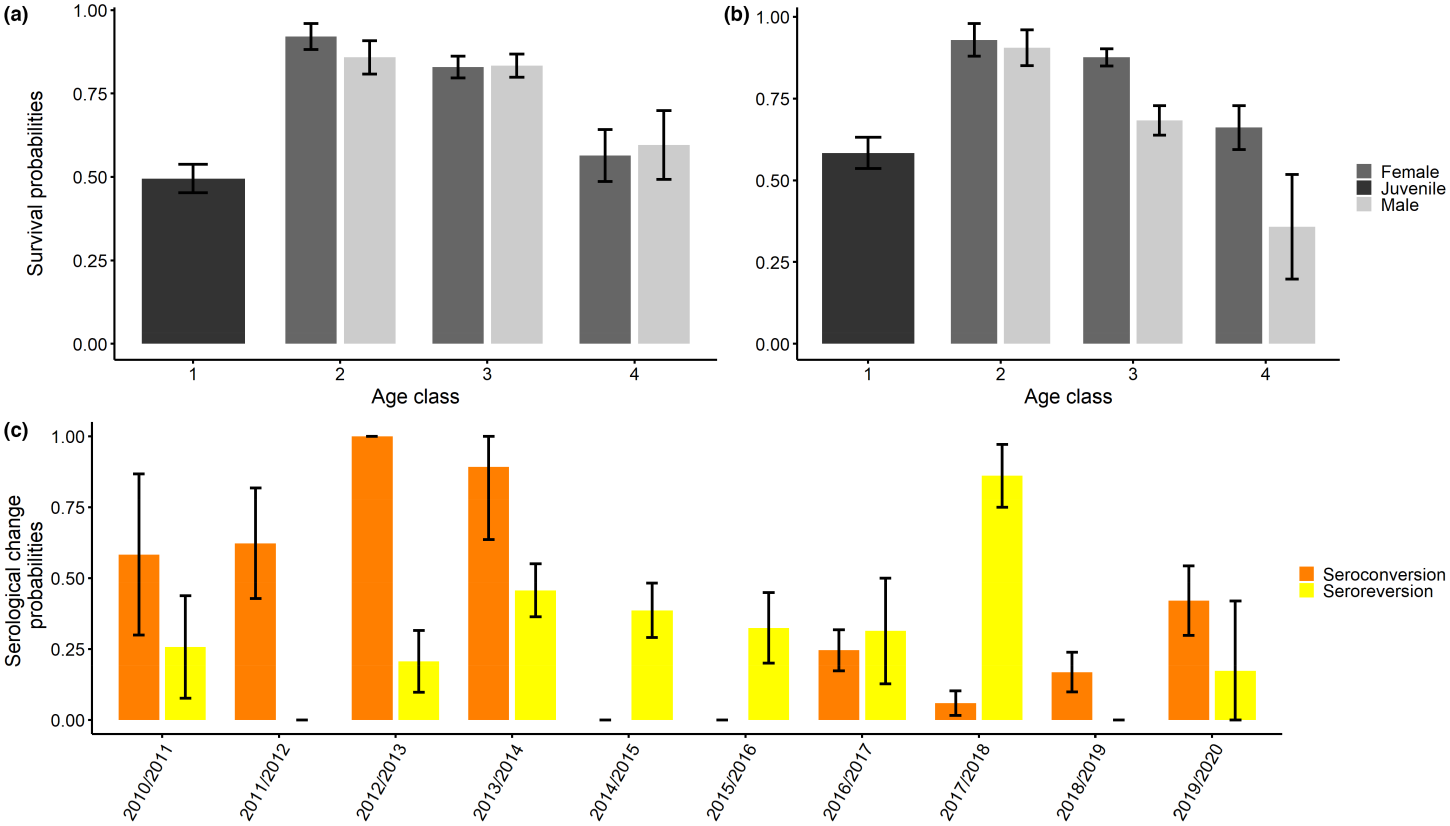
Annual survival and transition rates estimated from the CMR analyses conducted in both sites from the best model. Based on a previous demographic work, the sex effect was not considered on the survival for juvenile individuals (i.e. ψ = a2) (Gaillard et al., 1997). In the other age classes, survival is represented in dark grey for females and in light grey for males in TF (a) and CH (b). The seroconversion and seroreversion rates were estimated from the best model including the time on the annual serological rates of change (c). Error bars for each bar plot represent the standard errors.

There was no evidence of an association between the annual survival probabilities and the serological status of roe deer both in TF and CH (see Table S5). In TF, annual survival was low for juveniles (0.495; SE: 0.042), high for 2–3‐year‐old individuals (females: 0.921, SE: 0.039 and males: 0.858, SE: 0.050) and then decreased with increasing age, notably for individuals older than 4 years (females: 0.829, SE: 0.032 and males: 0.833, SE: 0.035), and even more for the class including the oldest individuals (more than 9 years old) (females: 0.564, SE: 0.078 and males: 0.595, SE: 0.103) (Figure 4a). In CH, survival was also lowest for juveniles (0.584, SE: 0.048), increased for 2–3‐year‐old individuals (females: 0.929; SE: 0.050; males: 0.906; SE: 0.055) and then decreased for individuals older than 4 years (females: 0.876, SE: 0.026; males: 0.683; SE: 0.045) and even more for the oldest individuals (more than 9 years old) (females: 0.661; SE: 0.067; males: 0.358; SE: 0.159) (Figure 4b).

### 3.5 Annual rates of change in serological status

Annual variation in serological status was influenced by year, with no evidence of differences between locations, sex or age classes (Table 1a). Overall, the average annual seroconversion rate (0.185, SE: 0.061) was close to the average annual seroreversion rate (0.196, SE: 0.068). In addition, seroconversion rates were higher than seroreversion rates in the early years of monitoring (2010–2014) and then again during the last year (2019–2020) (Figure 4c).

### 3.6 Within‐year dynamics of anti‐ *Bbsl* antibody levels

**FIGURE 5:**
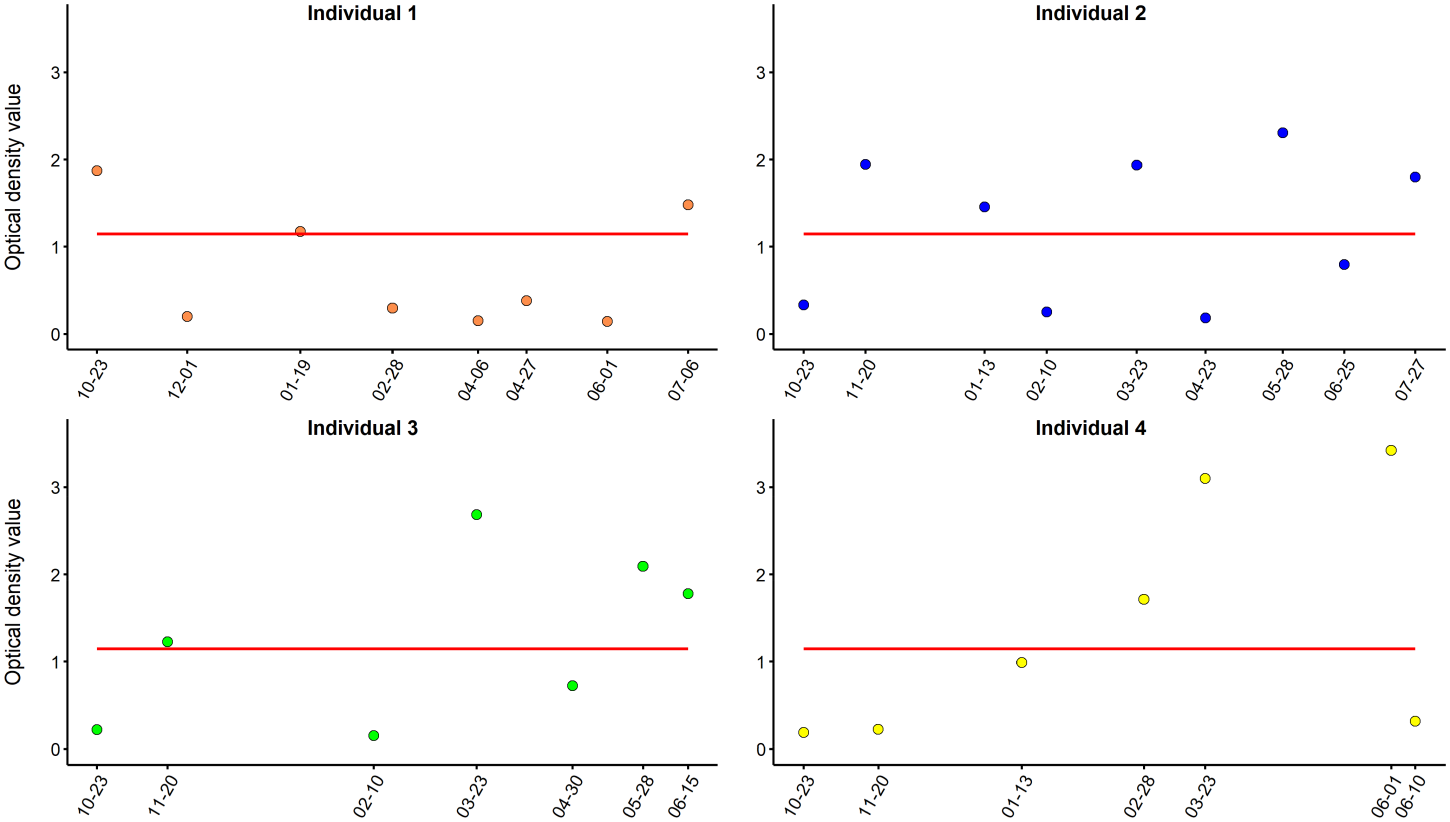
Dynamics of antibody levels against *Bbsl* were assessed via repeated sampling of four individuals in the experimental platform of GA from October 2020 to July 2021. OD values are represented by orange points (individual 1), blue points (individual 2), green points (individual 3), yellow points (individual 4). The red full line represents the seropositivity threshold (OD = 1.146).

To investigate the intra‐annual persistence of antibody levels in roe deer, four females from the experimental platform of GA allowed us to obtain some information on the intra‐annual persistence of antibody levels in roe deer. These four females have been sampled repeatedly at least seven times from October 2020 to July 2021. They were from age class 3 and 4 (individuals 1 and 4 were from age class 3 and the two others from age class 4). For all four individuals, OD values varied over short time scales, showing little stability between successive months (Figure 5). Over the 9‐month study period, we observed highly dynamic changes in anti‐*Bbsl* antibody levels, with 1–4 seroconversion events and 1–3 seroreversion events per individual when considering a threshold comparable to that of the two long‐term monitored populations.

## 4 DISCUSSION

### 4.1 *Bbsl* exposure and host seroconversion and seroreversion rates

In this study, we used a repeated sampling of individual roe deer analysed in a CMR framework to infer the dynamics of their serological status against an infectious agent of public health interest. For this purpose, we adapted to roe deer an ELISA originally developed for human samples to detect antibodies against the agent of Lyme disease and determined a threshold for seropositive animals using a Gaussian mixture model applied to the distribution of OD from the ELISA. Used together, those tools highlighted the potential of roe deer as a sentinel to track exposure risk to *Bbsl* across space and time.

When plotting the distribution of the OD values, we found a bimodal pattern with an overlap between the curves. Determining the serological status against *Bbsl* using ELISA OD values has previously been done in other wildlife species, for example in a cliff‐nesting seabird species, the black‐legged kittiwake (*Rissa tridactyla*) (Chambert et al., 2012). In that case, the distribution of OD values showed two largely non‐overlapping curves, one representing the seropositive individuals (i.e. high optical density values) and the other representing the seronegative individuals (i.e. low OD values). The non‐overlapping curves may then have been due to a relatively clear difference in the history of exposure of individuals to infected ticks. Kittiwakes are long lived, highly faithful to their breeding site and the *Bbsl* tick vector to which they are exposed is strongly heterogeneously distributed in space. In that host species, highly stable OD values were recorded between years (Staszewski et al., 2007), in contrast to our results in roe deer.

In roe deer, we estimated a high repeatability of the ELISA assay, thus uncertainty in the test result is probably not the cause of the substantial overlap of OD values we reported. One alternative explanation could be the low temporal persistence of antibodies in that species and high exposure to *Bbsl*‐infected ticks, leading to a sizeable proportion of individuals being at any time in transition between the seronegative and seropositive state, or the reverse. Individuals with intermediate antibody levels could either be currently mounting a specific humoral immune response or their antibody level may be waning after an earlier exposure. The intermediate antibody levels recorded for many individuals could also be related to the effectiveness of the innate immunity, linked to its non‐competence as a host for *Bbsl* (Jaenson & Tälleklint, 1992; Telford et al., 1988). Sera collected from red, sika, mule, roe and, lately, white‐tailed deer have been reported to kill different *Borrelia* genospecies (Bhide et al., 2005; Kurtenbach et al., 1998; Nelson et al., 2000; Pearson et al., 2023; Ullmann et al., 2003), but little information is available on how innate and acquired immune responses interact in those host species (Bhide et al., 2004, 2005). The complex processes involved in complement evasion by *Bbsl* spirochetes, with the specific involvement of molecules in interfering with acquired immunity (Dulipati et al., 2020; Kraiczy et al., 2002), suggest that the topic is not simple. A key issue related to the potential use of deer as sentinels would thus be to explore how the history of individual exposure to infected ticks, considering different *Bbsl* spirochetes, affects detectable anti‐*Bbsl* antibody levels.

Although the sample size is limited, the intra‐annual monitoring of the serological status of captive roe deer also suggests a short‐term persistence of detectable antibody anti‐*Bbsl* levels over a few weeks. This result differs from those reported in white‐tailed deer, in which antibody responses mounted against *Borrelia burgdorferi* sensu stricto were found to persist for at least 10 weeks post‐infection (Luttrell et al., 1994). The difference between results could be due to the protocols used. In Luttrell et al. (1994), deer were experimentally infected by injection of *Bbsl*, whereas in the present study, deer had been naturally exposed. The low persistence of the anti‐*Bbsl* antibodies in roe deer indicates that serological data could provide information on infection status close to sample collection. The serological monitoring of roe deer could thus provide a snapshot of the infected tick risk in the weeks preceding the sampling.

### 4.2 Epidemiological and demographical processes

Modelling annual survival and transition rates between anti‐*Bbsl* serological status using multi‐event CMR modelling in roe deer, we detected strong effects of age and sex on survival patterns and a substantial temporal variation in seroconversion and seroreversion in both populations. Similar patterns of an increase of annual survival between young and middle‐aged individuals and a decline thereafter have already been reported for the same populations, including earlier than this study (Gaillard et al., 1993). Senescence of survival may be related to tooth wear or immunosenescence (Cheynel et al., 2017). Annual survival was lower in males than in females in TF, while males had an overall lower survival in CH.

Regarding temporal changes in yearly rates of change in serological status, high seroconversion rates were detected early in the study period, as well as high overall seroreversion rates (Figure 4c). These results were congruent with the relative similarity of the temporal patterns of prevalence observed between sites, notably lower seroprevalences later in the time series. As the two study sites are located 800 km apart and in different forests in terms of productivity, such synchronized variation between sites could be due to common environmentally driving factors, such as changes in climate (Dautel et al., 2008; Wongnak et al., 2022), tick density (Tälleklint & Jaenson, 1996) or rodent host density (Perez et al., 2016), but also to differences in deer susceptibility. A recent study that explored the variability in tick burden on young roe deer fawn at TF over a 20‐year period showed a clear between‐year variability in mean tick burden, without identifying any correlation with environmental factors (Bariod et al., 2022). A common pattern between sites is the decline of roe deer population reproductive performance over time (Plard et al., 2014), which might contribute to shape the patterns of seroprevalence via potential effects on immunity. Individuals need to allocate more energy for reproductive performance which may limit the energy used for the immune responses.

No effect of sex or age was detected on serological transitions. In roe deer, the home range size is fairly similar between sexes, which may limit potential sex‐associated differences in exposure to ticks. Several previous studies have reported that male roe deer were more in contact with ticks than females (Kiffner et al., 2010; Vázquez et al., 2011; Vor et al., 2010). However, this difference between sexes may be observable only in spring and summer, when ticks are more active (Gray et al., 2009; Vázquez et al., 2011) but not in winter, when roe deer were captured in our study. A key issue is also that the abundance of ticks is not necessarily related to the abundance of infected ticks, which depends on the transmission of the bacteria among competent species. The results of the CMR modelling approach highlighted the benefit of accounting for demographic and epidemiological processes when analysing time series of serological status data. Complex dynamics are nevertheless likely involved in the infection and antibody responses within the communities of hosts of *Bbsl* and further work should consider how various key compartments may be affecting the dynamics.

### 4.3 Roe deer as a sentinel for the surveillance of exposure risk to Lyme disease agent?

Our findings suggest that roe deer serological surveys could provide information on their local exposure to infected ticks over the recent time period before sampling. The main deer hunting season in Europe is in winter, which could facilitate access to samples at that time to explore exposure to infected ticks at broader spatial and temporal scales. The seasonality of tick abundance often shows two peaks in temperate regions, one in spring and another in autumn (Hauser et al., 2018; Schulz et al., 2014). The highest risk of exposure for humans corresponds to the spring peak of tick density (Fu et al., 2021), when roe deer samples for serology may be difficult to get. Detecting the density of active ticks positive for *Bbsl* in winter via roe deer serology could nevertheless be useful if those are related to the acarological risk, that is the density of infected ticks in spring. Several studies have shown that the period of tick activity can be extended during mild winters, which are expected to occur more frequently in the context of climate change (Dautel et al., 2008). The survey of the serological status of roe deer hunted in winter could thus represent a useful snapshot of the density of infected ticks before the peak of tick activity in spring. The changes in serological transition rates that we detected suggest that the serology of roe deer could be sensitive enough to assess annual variations in densities of infected ticks. In addition, our results provide evidence that neither sex nor age of individual roe deer has to be accounted for to obtain reliable information about the exposure to infected ticks. This result suggests that the sampling would not be biased depending on the age/sex of the hunted roe deer and that all samples of hunted roe deer could be considered in the context of large‐scale monitoring.

Another criterion to evaluate whether a given species could be a good sentinel is the impact of the serological status on the survival of the sentinel. If seropositive individuals die at a higher rate than seronegative ones, sampling would be biased toward uninfected individuals and the risk of exposure to *Bbsl* would be underestimated. As expected, we did not detect any association between the serological status against *Bbsl* of roe deer and their annual survival. This is consistent with previous results on other species not showing such associations (Chambert et al., 2012; Voordouw et al., 2015), and with the facts that deer do not develop symptoms and that the bacteria are killed by the immune complement (Isogai et al., 1994).

Overall, our combined epidemiological and demographic analysis highlights the diverse benefits that can be gained from long‐term capture‐mark‐recapture monitoring integrating complementary data and sampling collection in a widespread and abundant species.

## 5 CONCLUSION

Despite the broad use of cross‐sectional serological surveys to assess the exposure of wild vertebrate populations to infectious agents, little information is available on the temporal dynamics of antibody response of individuals, which may limit the interpretation of the reported patterns. Using a modified commercially available immunoassay to detect antibodies against Lyme disease agent and blood samples collected in two wild roe deer populations monitored over 10 years, we conducted multi‐event capture–mark–recapture modelling on individual histories of capture and serological status to estimate population‐, sex‐ and age‐class‐specific rates of seroconversion and seroreversion while accounting for recapture probability and survival. The seropositivity threshold was determined using a mixed Gaussian model on the distribution of optical density values from the immunoassay. The high seroprevalences and seroconversion rates that were estimated for the first years of the monitoring in both populations suggest a higher level of exposure to infected tick bites at that time. The relatively high rates of seroreversion estimated over the whole study period indicated the short‐term persistence of antibodies against *Bbsl* in the considered host species. This result was congruent with highly dynamical antibody levels obtained for a few roe deer that could be sampled repeatedly a few weeks apart at a third site. Age and sex effects on annual survival were found as in previous studies conducted in the two populations, while no association between serological status against *Bbsl* and survival probability was detected. The large overlap in OD values between potentially seropositive and seronegative individuals was likely related to the dynamic nature of the level of antibodies in the deer. The results highlight the potential usefulness of roe deer as a sentinel for tracking exposure to the agent of Lyme disease. They also highlight the value of capture–mark–recapture sampling and analyses of serological data for wildlife populations exposed to infectious agents of relevance to wildlife epidemiology and human health.

## AUTHOR CONTRIBUTIONS

**Valentin Ollivier:** Conceptualization (equal); formal analysis (equal); investigation (equal); methodology (equal); validation (equal); visualization (equal); writing – original draft (equal). **Rémi Choquet:** Conceptualization (equal); formal analysis (equal); methodology (equal); software (equal); supervision (equal); writing – review and editing (equal). **Amandine Gamble:** Conceptualization (equal); formal analysis (equal); investigation (equal); methodology (equal); writing – review and editing (equal). **Matthieu Bastien:** Investigation (equal); supervision (equal); writing – review and editing (equal). **Benoit Combes:** Investigation (equal); writing – review and editing (equal). **Emmanuelle Gilot‐Fromont:** Investigation (equal); resources (equal); writing – review and editing (equal). **Maryline Pellerin:** Investigation (equal); resources (equal); writing – review and editing (equal). **Jean‐Michel Gaillard:** Investigation (equal); resources (equal); writing – review and editing (equal). **Jean‐François Lemaître:** Investigation (equal); resources (equal); writing – review and editing (equal). **Hélène Verheyden:** Investigation (equal); resources (equal); supervision (equal); writing – review and editing (equal). **Thierry Boulinier:** Conceptualization (equal); investigation (equal); methodology (equal); supervision (equal); writing – review and editing (equal).

## FUNDING INFORMATION

This study was conducted in the context of a PhD supported by a CIFRE convention between ANRT, CNRS and INRAE. Support from FEDER is acknowledged.

## CONFLICT OF INTEREST STATEMENT

The authors declare no conflicts of interest.

## Supporting information


Appendix S1

## Data Availability

Data are available on zenodo at https://doi.org/10.5281/zenodo.7506275.
