# Supplementary material for: Temporal dynamics of antibody level against Lyme disease bacteria in roe deer: Tale of a sentinel?
Source: Ecol Evol. 2023 Aug 17;13(8):e10414. doi: 10.1002/ece3.10414 (PMC10433119; doi:10.1002/ece3.10414)
Supplement: Supplementary file 1 — Appendix S1 [file ECE3-13-e10414-s001.docx]

**Appendices**

**Table S1.** Table showing the number of individuals sampled each year according to their serological status for each year between 2010 and 2020 for TF (A) and CH (B).

|  | TF | | CH | | |  |
| --- | --- | --- | --- | --- | --- | --- |
|  | **Seronegative** | **Seropositive** | | **Seronegative** | **Seropositive** | |
| 2010 | 47 | 31 | | 3 | 3 | |
| 2011 | 50 | 18 | | 68 | 11 | |
| 2012 | 57 | 16 | | 61 | 6 | |
| 2013 | 43 | 35 | | 30 | 40 | |
| 2014 | 36 | 33 | | 32 | 38 | |
| 2015 | 58 | 18 | | 68 | 10 | |
| 2016 | 68 | 13 | | 60 | 19 | |
| 2017 | 70 | 17 | | 77 | 26 | |
| 2018 | 68 | 8 | | 57 | 1 | |
| 2019 | 62 | 13 | | 51 | 10 | |
| 2020 | 36 | 14 | | 55 | 17 | |

| A | Females | | | | Males | | | |
| --- | --- | --- | --- | --- | --- | --- | --- | --- |
|  | **1** | **2** | **3** | **4** | **1** | **2** | **3** | **4** |
| 2010 | 16 | 10 | 12 | 9 | 14 | 2 | 7 | 8 |
| 2011 | 9 | 11 | 5 | 10 | 19 | 4 | 5 | 5 |
| 2012 | 18 | 7 | 4 | 10 | 17 | 4 | 6 | 7 |
| 2013 | 9 | 10 | 13 | 12 | 8 | 11 | 8 | 7 |
| 2014 | 9 | 5 | 9 | 9 | 10 | 8 | 16 | 3 |
| 2015 | 20 | 5 | 12 | 3 | 11 | 6 | 15 | 4 |
| 2016 | 13 | 3 | 15 | 9 | 19 | 3 | 13 | 6 |
| 2017 | 18 | 8 | 16 | 10 | 15 | 3 | 8 | 9 |
| 2018 | 18 | 9 | 4 | 10 | 16 | 7 | 9 | 3 |
| 2019 | 10 | 9 | 6 | 11 | 16 | 5 | 11 | 7 |
| 2020 | 6 | 12 | 5 | 6 | 5 | 5 | 3 | 8 |

**Table S2.** Tables showing the numbers of individuals sampled by sex and age class for each year between 2010 and 2020 for TF (A) and CH (B).

| B | Females | | | | Males | | | |
| --- | --- | --- | --- | --- | --- | --- | --- | --- |
|  | **1** | **2** | **3** | **4** | **1** | **2** | **3** | **4** |
| 2010 | 1 | 0 | 0 | 1 | 2 | 0 | 1 | 1 |
| 2011 | 10 | 12 | 14 | 6 | 10 | 12 | 8 | 7 |
| 2012 | 11 | 9 | 19 | 4 | 9 | 8 | 3 | 4 |
| 2013 | 12 | 7 | 16 | 9 | 13 | 6 | 4 | 3 |
| 2014 | 12 | 4 | 13 | 7 | 14 | 6 | 9 | 5 |
| 2015 | 15 | 4 | 12 | 12 | 17 | 7 | 5 | 6 |
| 2016 | 9 | 5 | 14 | 14 | 13 | 7 | 9 | 8 |
| 2017 | 18 | 14 | 14 | 10 | 21 | 11 | 14 | 1 |
| 2018 | 13 | 9 | 7 | 6 | 7 | 7 | 6 | 3 |
| 2019 | 9 | 6 | 10 | 7 | 16 | 3 | 9 | 1 |
| 2020 | 10 | 8 | 15 | 6 | 13 | 8 | 6 | 6 |

**Table S3.** GEMACO shortcuts used in the E-surge software to group individuals by age classes.

|  |  |  | **GEMACO Shortcut** |
| --- | --- | --- | --- |
| **For initial proportion (π), survival (s) and serological status change (ψ)** | **juv** | Shortcut which included the individuals ≤ 1-year-old | [a(1).REALAGE(1)] |
|  | **sad** | Shortcut which included the individuals 1<year old ≤3 | [a(2:3).REALAGE(1)&a(1:2).REALAGE(2)&a(1).REALAGE(3)] |
|  | **ad** | Shortcut which included the individuals 3<year old≤9 | [a(4:9).REALAGE(1)&a(3:8).REALAGE(2)&a(2:7).REALAGE(3)&a(1:6).REALAGE(4)&a(1:5).REALAGE(5)&a(1:4).REALAGE(6)&a(1:3).REALAGE(7)&a(1:2).REALAGE(8)&a(1).REALAGE(9)] |
|  | **old** | Shortcut which included the individuals > 9 year old | [a(10:16).REALAGE(1)&a(9:16).REALAGE(2)&a(8:16).REALAGE(3)&a(7:16).REALAGE(4)&a(6:16).REALAGE(5)&a(5:16).REALAGE(6)&a(4:16).REALAGE(7)&a(3:16).REALAGE(8)&a(2:16).REALAGE(9)&REALAGE(10:16)] |
|  | **ACtot** | Shortcut which included all age classes | [juv+sad+ad+old] |
| **Shortcut for recapture (p¸)** | **cjuv** | Juvenile individuals recaptured | [a(2).REALAGE(1)] |
|  | **cad** | Other age classes recaptured | [a(3:11).REALAGE(1)&REALAGE(2:16)] |

**Table S4.** Model selection for the five most supported multi-events model (A) and their GEMACO sentence used in the E-surge software (B). The general model included π(pop.a4.sex), s(pop.sex.f.a4.t), ψ(pop.a2.sex.f.t), p(pop.a2.t), σ (t), e(c), where π if the initial status proportions, s is the survival, ψ is the serological transition rate, p is the recapture rate, σ is whether the ELISA test was performed and e is the error on the serological assignment. For the effects, t denoted the year effect, a4 is the age effect for survival, as defined previously, f is the serological status effect, a2 is the age effect for the capture (i.e., 1 year after marking as juvenile *versus* the rest) and c is a constant effect. Model selections were conducted by varying the parameters that could affect the serostatus transitions. Among the parameters, we included the effect of year “time”, the effect of age class “juv, sad, ad and old” and. In addition, plus “+” correspond to an additive effect and the point (.) an interactive effect between parameters. The ELISA error is empty and correspond to a constant effect.

| **A** | **Initial states (π)** | **Survival (s)** | **Serological change (**ψ**)** | **Recapture (p)** | **Test perform (σ)** | **ELISA error (e)** | ***∆QAICc*** |
| --- | --- | --- | --- | --- | --- | --- | --- |
| **Model** |  |  |  |  |  |  |  |
| ψ=f.(t+pop) | a4 | pop.a2.sex | f.(t+pop) | pop.a2.t | t | c | *-* |
| ψ= f.t | a4 | pop.a2.sex | f.t | pop.a2.t | t | c | 0.1254 |
| ψ= f.(t +a4 + pop) | a4 | pop.a2.sex | f.(t +a4 + pop) | pop.a2.t | t | c | 2.1525 |
| ψ= f.(t + sex + pop) | a4 | pop.a2.sex | f.(t + sex + pop) | pop.a2.t | t | c | 2.2303 |
| ψ= f.(t + sex + pop) | a4 | pop.a2.sex | f.(t + sex + pop) | pop.a2.t | t | c | 2.2688 |

| **B** | **Initial states (π)** | **Survival (s)** | **Serological change (**ψ**)** | **Recapture (p)** | **Test perform (σ)** | **ELISA error (e)** | ***∆QAICc*** |
| --- | --- | --- | --- | --- | --- | --- | --- |
| **Model** |  |  |  |  |  |  |  |
| ψ= f.(t+pop) | REALAGE(1_3,4:13) | POP.[juv+SEX.[sad+ad+old]] | f.[time+POP] | firste+nexte.POP.[cjuv.t+cad.t] | time |  | *-* |
| ψ= f.t | REALAGE(1_3,4:13) | POP.[juv+SEX.[sad+ad+old]] | f.time | firste+nexte.CJUV.t+CAD.t] | time |  | 0.1254 |
| ψ= f.(t +a4 + pop) | REALAGE(1_3,4:13) | POP.[juv+SEX.[sad+ad+old]] | f.time+[juv+ sad+ad+old]+POP | firste+nexte. CJUV.t+CAD.t] | time |  | 2.1525 |
| ψ= f.(t + sex + pop) | REALAGE(1_3,4:13) | POP.[juv+SEX.[sad+ad+old]] | f. [time+ACtot] | firste+nexte. CJUV.t+CAD.t] | time |  | 2.2303 |
| ψ= f.(t + sex + pop) | REALAGE(1_3,4:13) | POP.[juv+SEX.[sad+ad+old]] | f. [time+SEX+ACtot] | firste+nexte. CJUV.t+CAD.t] | time |  | 2.2688 |

**Table S5.** Multi-event model selection to assess the impact of the serological status on survival probabilities.

**Model parameterization on survival probabilities**

| *Survival* | *Id parameters* | *Deviance* | *QAICc* | *ΔQAICc* |
| --- | --- | --- | --- | --- |
| s= pop.sex.a4.t | 318 | 5820.185 | 6456.185 | - |
| s= pop.sex.f.a4.t | 380 | 5793.668 | 6553.668 | 97.483 |

**Initial states (π):**

**
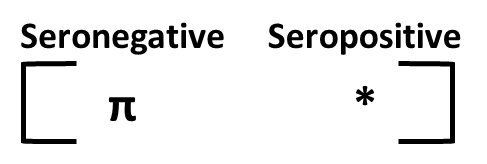
**

**Biological processes:**

**Step 1:** Survival (s)

**
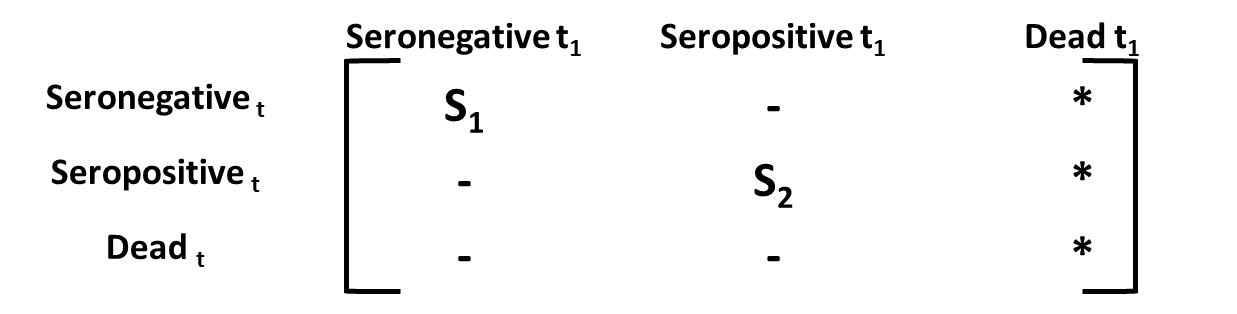
**

**Step 2 :** Serological change (Ѱ)

**
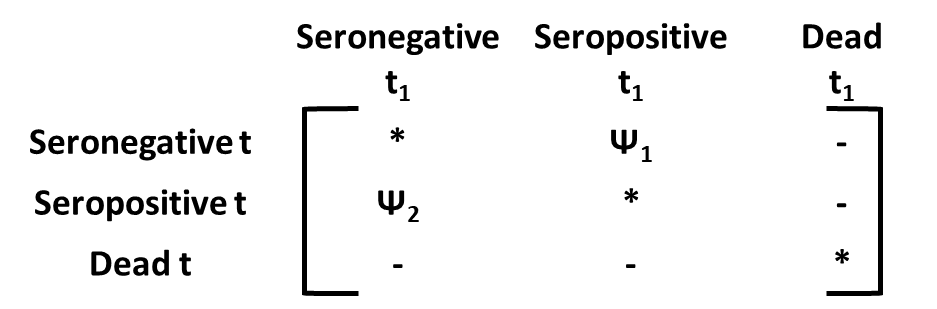
**

**Observational processes**

**Step 1:** Recapture (p)


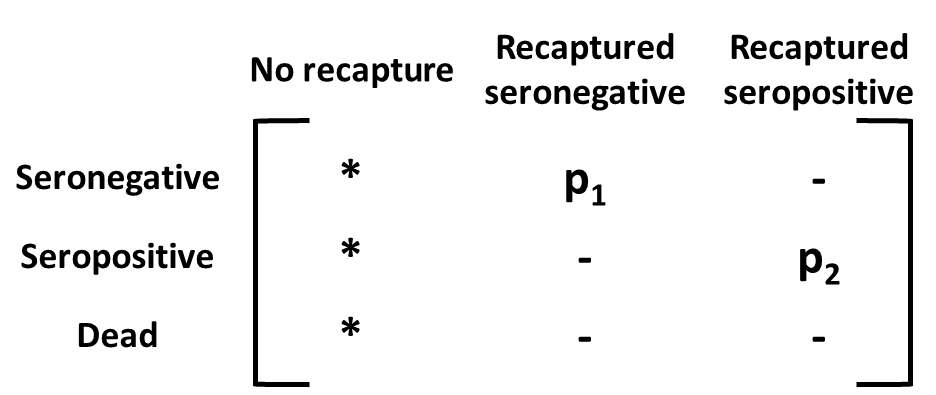


**Step 2:** Test performed (σ)

**
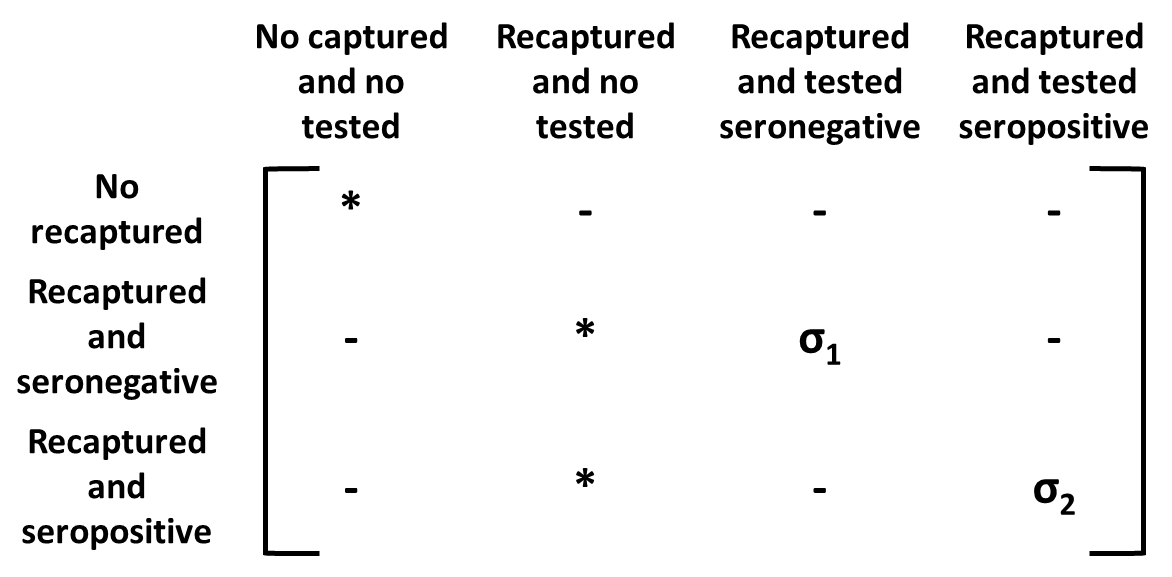
**

**Step 3:** ELISA error assignment (e)

**
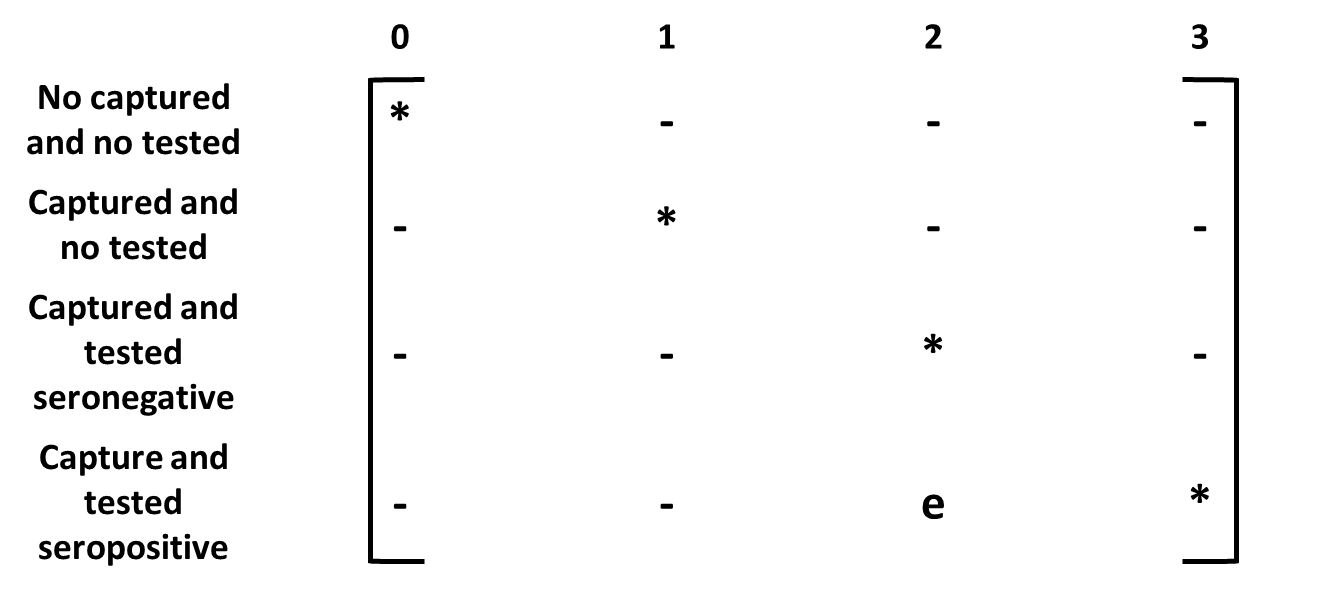
**

**Figure S1**. GEPAT matrix used in the E-surge software to create the multi-event models structures in both populations. In the matrix, the “-” symbol means a null probability and the “*” indicates the complementary probability.

**
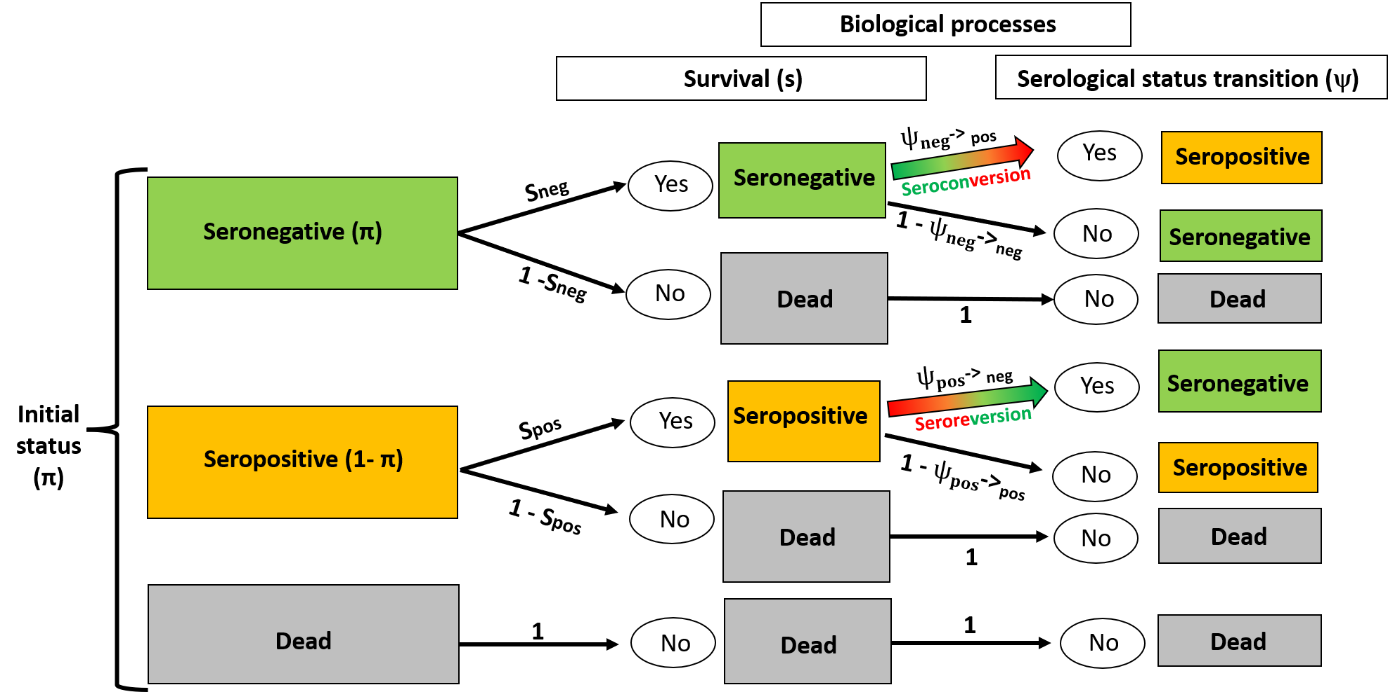
**

**
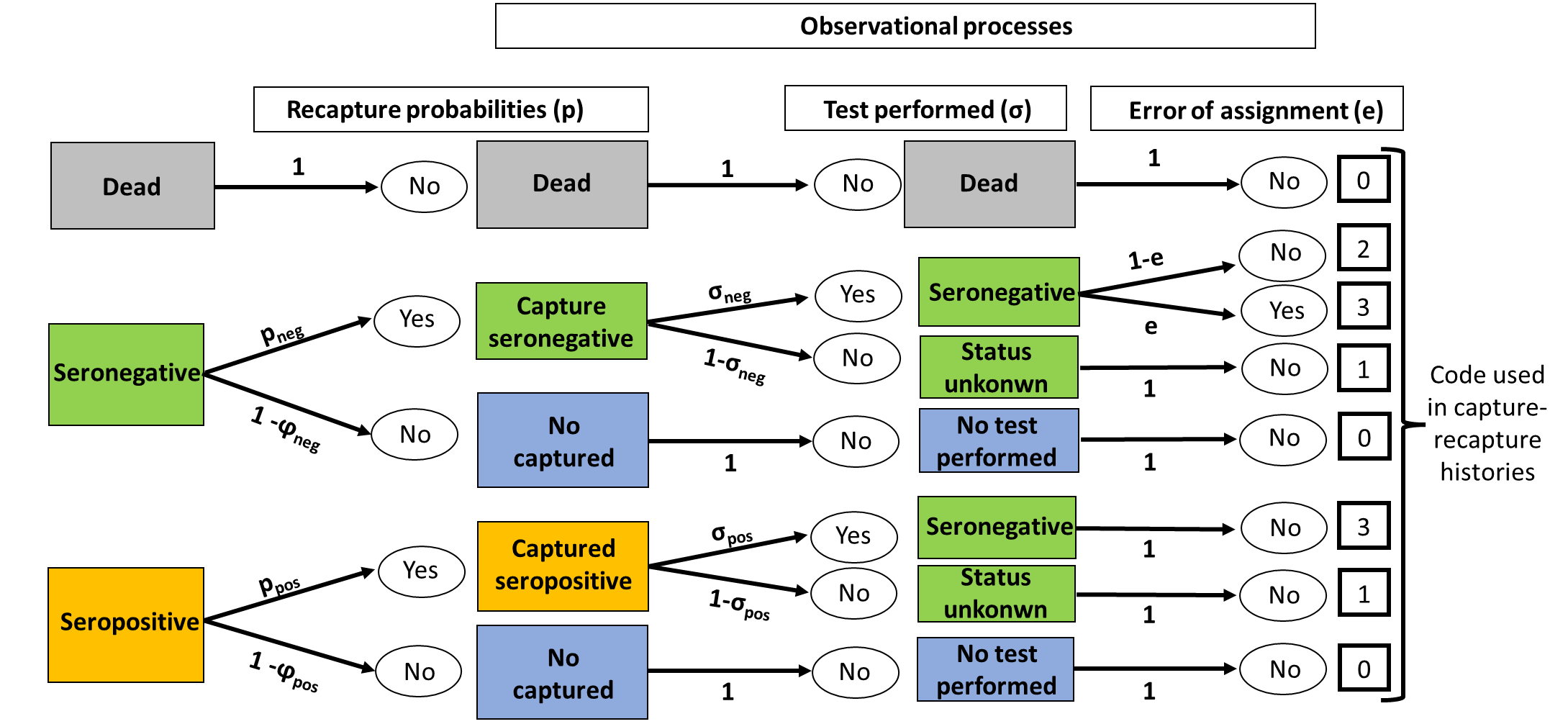
**

**Figure S2**. Illustration of the structure of the multi-event model used with the tree of probabilities associated with each successive matrices in biological and observational processes.

**
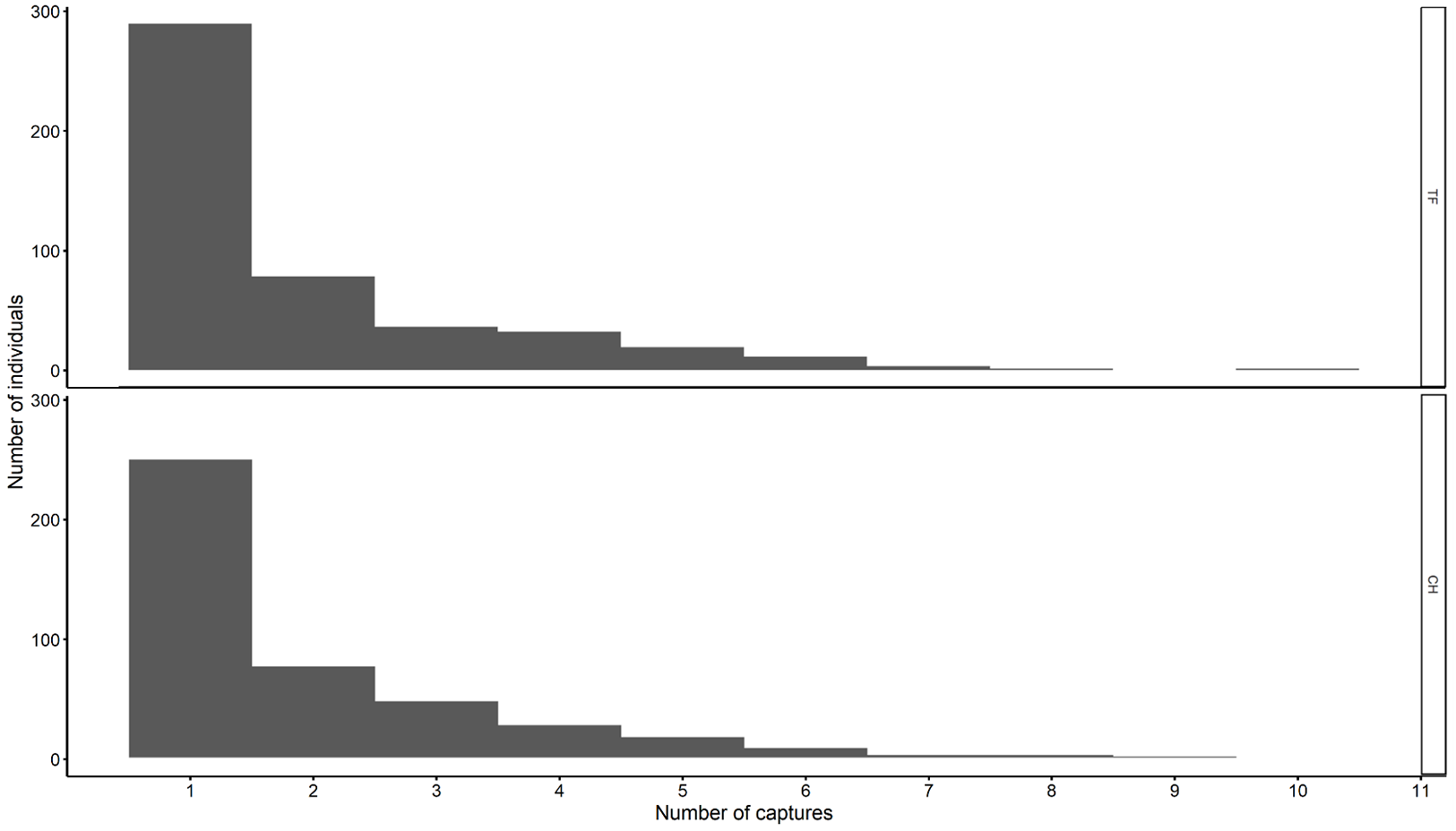
**

**Figure S3**. Distribution of the number of individuals according to their number of captures.
